# Improving the Stability of Lycopene from Chemical Degradation in Model Beverage Emulsions: Impact of Hydrophilic Group Size of Emulsifier and Antioxidant Polarity

**DOI:** 10.3390/foods9080971

**Published:** 2020-07-22

**Authors:** Jinhyuk Kim, Seung Jun Choi

**Affiliations:** 1 Department of Food Science and Technology, Seoul National University of Science and Technology, Seoul 01811, Korea; rla994@seoultech.ac.kr; 2 Department of Interdisciplinary Bio IT Materials, Seoul National University of Science and Technology, Seoul 01811, Korea

**Keywords:** amphiphilic antioxidants, emulsions, interfacial film, lipophilic antioxidants, lycopene

## Abstract

The chemical stability of the lipophilic bioactives encapsulated in emulsions can be influenced by emulsion droplet interfacial characteristics as well as by the ability of antioxidants incorporated in emulsion to prevent the degradation of the encapsulated compounds. Therefore, this study evaluated the effects of the interfacial characteristics of emulsions and the polarity of antioxidants on the storage stability of lycopene in emulsions. Emulsions with 5% (*w*/*w*) oil containing lycopene (30 µmol/kg emulsion) were prepared using a series of polyethylene glycol acyl ether-type emulsifiers through microfluidization. Change in lycopene content in emulsions was monitored by high performance liquid chromatography. Our findings show that the hydrophilic group size (or length) of emulsifiers and the emulsifier concentration at the interfacial film play a role, albeit minor, in controlling the storage stability of lycopene encapsulated in emulsions. Lipophilic (*tert*-butylhydroquinone (TBHQ)) and amphiphilic (lauryl gallate) antioxidants similarly improved the storage stability of lycopene in emulsions from acid- and radical-mediated degradation, independent of the characteristics of interfacial films of emulsions. However, TBHQ inhibited the degradation of lycopene in emulsions more effectively than lauryl gallate under conditions intended to accelerate the acid-mediated degradation of lycopene. Therefore, our findings can provide helpful information about what type of emulsifiers and antioxidants can be chosen for preparing food emulsions capable of maximizing the stability of lycopene encapsulated therein.

## 1. Introduction

Carotenoids are natural pigments responsible for the red, orange, and yellow colors of several vegetables and fruits. They are all derivatives of tetraterpenes, and they are classified into xanthophylls and carotenes [1,2]. The antioxidant activity of carotenoids can help prevent tissue damage caused by several oxidative stresses [3,4]. Among these carotenoids, lycopene represents an attractive dietary target for the beneficial health effects because of their antioxidant activities [5,6], the protective effect against cancer and prostate tumors [7], and cardiovascular disease [8]. This suggests that lycopene can provide potential health benefits in terms of the prevention/reduction of cancers and cardiovascular diseases [9]. For these reasons, lycopene has generated high levels of interest regarding its incorporation into general and functional food products.

The incorporation of carotenoids including lycopene in a variety of food products is not easy [10] because carotenoids are not water-soluble [3]. Since oil droplets containing high levels of carotenoids are dispersed into the aqueous phase in emulsion systems, oil-in-water emulsions are very effective at increasing the solubility of carotenoids. Several studies have found that the encapsulation of carotenoids into emulsions is an effective way to incorporate carotenoids into food and beverage products at high levels [11,12,13]. The incorporation of carotenoids into oil-in-water emulsions can also improve their bioaccessibility [14,15]. The chemical stability of carotenoids against oxidative degradation can be improved since the interfacial films of oil droplets can isolate carotenoids from water-soluble materials that include acids, transition metals, and radicals, which are able to induce the oxidative degradation of carotenoids [16]. In addition, the simple isolation of carotenoids by interfacial films from water-soluble materials (prooxidants) is able to accelerate carotenoid degradation, and the interfacial film, as physical barrier, can prevent and interrupt the direct interaction of oxidation-promoting compounds in the aqueous phase with carotenoids in oil droplets [17]. These findings suggest that the properties of interfacial film could be important determinants in controlling the chemical stability of carotenoids encapsulated into emulsion droplets. Additionally, these observations suggest that the careful selection of emulsifiers that are constituent components of interfacial films could improve the chemical stability of carotenoids encapsulated into emulsions [18]. However, despite the isolation and protection of carotenoids from prooxidants in the aqueous phase by interfacial membranes, the concentration of carotenoids may gradually decrease over the storage period. To increase the chemical stability of carotenoids in emulsions, the addition of antioxidants to emulsions can be helpful. The addition of lipophilic antioxidants (coenzyme Q10, eugenol, and α-tocopherol, etc.) to the oil phases of emulsions [19,20] and the addition of hydrophilic (ascorbic acid and gallyl derivatives, etc.) to their aqueous phases were effective in reducing the extent and rate of the oxidative degradation of carotenoids in emulsions [19,21]. Sometimes the utilization of lipophilic and hydrophilic antioxidants in combination may be more effective than using individual components and sometimes it may not [21].

Compared with hydrophilic (water-soluble) antioxidants, hydrophobic (oil-soluble) ones are more effective in reducing the oxidation of emulsified oils [22,23,24] and preventing lipophilic functional compounds encapsulated into oil droplets from oxidative and chemical degradation [21]. However, according to recent studies [25,26], the efficiency of antioxidants in emulsions increases when their hydrophobicity (alkyl chain length) increases until a critical length showing the maximum efficiency is obtained. Antioxidants having alkyl chains longer than a critical length have decreased efficiency. When antioxidants have adequate hydrophilic-lipophilic balance values, they may be concentrated in the interfacial region of emulsions as the main site of lipid oxidation and degradation of lipophilic functional compounds. However, the strongly hydrophobic antioxidants may be located in the oily core of oil droplets in emulsions and their antioxidant efficiency decreases because of their physical location far from the interfacial region. Therefore, in this work, the effects of the interfacial characteristics (the hydrophilic group size (or length) of emulsifiers and the emulsifier concentration (density) at the interfacial films) of emulsions on the chemical stability of lycopene were evaluated. To determine the effects of the polarity of antioxidants, the effects of the incorporation of lipophilic and amphiphilic antioxidants into emulsions on the storage stability of lycopene encapsulated into emulsions were also determined. The findings in the current study will help in structuring effective emulsion-based encapsulation and delivery systems that can increase the chemical stability of lycopene during long-term storage.

## 2. Materials and Methods

### 2.1. Materials

**Figure 1:**
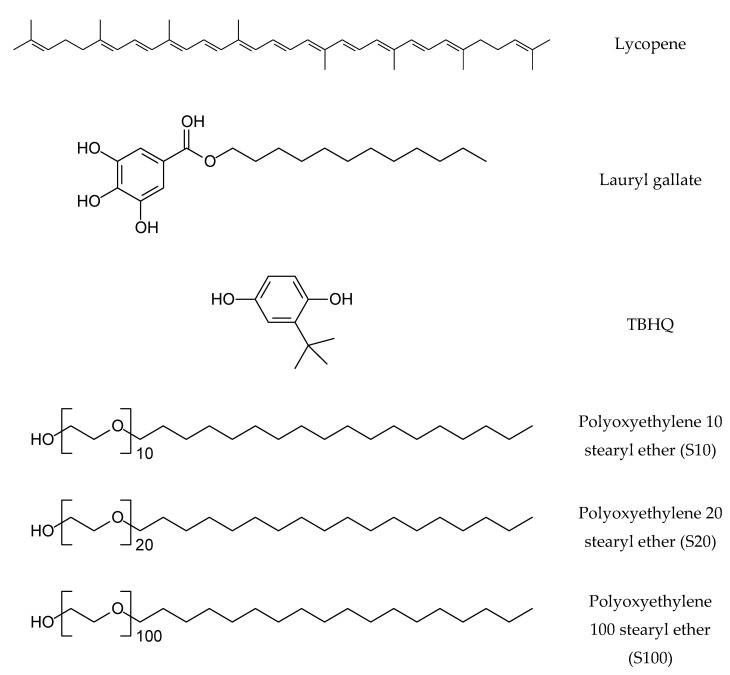
Molecular structures of lycopene, lauryl gallate, TBHQ, and polyethylene glycol acyl-type emulsifies used in this study.

Lauryl gallate, tert-butylhydroquinone (TBHQ), and 2,2′-azobis (2-methylpropionamidine) dihydrochloride (AAPH) were purchased from Sigma-Aldrich (St. Louis, MO, USA). Polyethylene glycol acyl ether-type emulsifiers (polyoxyethylene 10 stearyl (S10), polyoxyethylene 20 stearyl (S20), and polyoxyethylene 100 stearyl (S100) ethers), nonionic surfactants with hydrophilic groups compromising various numbers of oxyethylene groups, were also purchased from Sigma-Aldrich. The molecular structures of lycopene, lauryl gallate, TBHQ, and emulsifiers used in this work are presented in Figure 1. Lycopene (LycoVit^®^ Dispersion 10%) and medium chain triglyceride (Delios S) were purchased from BASF (Ludwigshafen, Germany). According to the manufacturer of LycoVit^®^ Dispersion 10%, it was oily dispersion containing lycopene in sunflower oil and its purity was 11.2% (*w*/*w*). All chemical reagents were of analytical grade.

### 2.2. Emulsion Preparation

In order to prepare the oil phase of the emulsions, lycopene was dissolved in medium chain triacylglycerol (MCT) to a final concentration of 0.3 mg/g MCT. The aqueous phase was prepared by dissolving emulsifiers in a 10 mM phosphate buffer (pH 7) at predetermined concentrations. Emulsions were prepared by mixing 5% oil phase with 95% (*w*/*w*) aqueous phase (30 µmol lycopene/1 kg emulsion). Coarse emulsions were prepared by mixing the oil and aqueous phases together using a high-speed blender (T18 Basic Ultra-Turrax, Ika, Staufen, Germany) at 11,000 rpm for 2 minutes at room temperature. For droplet size reduction, the coarse emulsions were homogenized with five passes through a microfluidizer (MN400BF, Micronox, Seongnam, Korea) at 100 MPa. Then, the pH of every emulsion sample was adjusted to pH 7 and 3. All emulsions were stirred by magnetic stirrer (MS-17GB, Jeio Tech., Daejeon, Korea) at 300 rpm at the desired pH for at least 30 minutes under nitrogen before storing them in darkness at 25 °C to prevent any potential degradation of lycopene by light.

In order to minimize any effect of micelles on the stability of lycopene encapsulated in emulsions, the minimum emulsifier concentrations (MEC) of emulsifiers were determined to prepare emulsions having high-stability emulsions with, if possible, the smallest droplet size using an MCT [27]. Emulsions were prepared by homogenizing the MCT (5%, *w*/*w*) as the oil phase and phosphate buffer solutions (95%, *w*/*w*) containing emulsifiers in a range from 0.1 to 14.0 mM. When emulsions were prepared at over the certain concentration of emulsifiers, droplet diameter did not decrease further with the increase in emulsifier concentration from the certain concentration of emulsifiers. It indicated that the droplet size was limited by the maximum amount of disruptive energy that could be generated by a microfluidizer. The certain concentration of emulsifier could be the minimum concentration of emulsifier required to produce the smallest droplet under the homogenization conditions applied in this study and this emulsifier concentration was defined to MEC.

To determine the effect of free radicals on the stability of lycopene in emulsions, AAPH solution was added to the emulsion samples up to the concentration of 20 µmol/kg emulsion. AAPH is a water-soluble azo compound which is used as a free radical-generator. It is often used in the study of lipid oxidation and the characterization of antioxidants [28]. Carbon radicals, the result of AAPH decomposition may react with molecular oxygen to yield peroxyl radicals. To study how the chemical stability of lycopene was affected by the polarity of antioxidants, antioxidants (lauryl gallate as an amphiphilic antioxidant and TBHQ as a lipophilic antioxidant) was added to the emulsions up to the concentration of 15 µmol/kg emulsion.

### 2.3. Droplet Size Measurement

The mean emulsion droplet diameters were measured using a static light scattering instrument (BT-9300ST; Bettersize Instruments, Dandong, China). To minimize the effect of multiple scattering, emulsion was diluted with a 10 mM phosphate buffer solution at the same pH of the sample to a droplet concentration of approximately 0.005% (*w*/*w*) and stirred gently throughout the measurements to ensure their homogeneity. The particle size data are reported as the volume-length mean diameter, $d_{43}=\sum n_{i}\cdot d_{i}^{4}/\sum n_{i}\cdot d_{i}^{3}$, where $n_{i}$ is the number of particles with diameter $d_{i}$.

### 2.4. Measurement of Lycopene Concentration

Lycopene concentrations in emulsions were determined by first vigorously vortexing 3 g of emulsion with 3 g of dichloromethane for 3 minutes. The mixture was then centrifuged at 1800× *g* for 10 minutes at 25 °C, and the solvent layer was collected. Quantification of lycopene in emulsions was performed by using an Agilent 1100 HPLC system (Palo Alto, CA, USA) following the previous study [29] with slight modification. Lycopene was separated with a Triart C18 column (250 mm × 4.6 mm × 5 μm, YMC, Tokyo, Japan) with a mobile phase (methanol:acetonitrile = 90:10 (*v*/*v*)). The flow rate and column temperature were 1 mL/min and 30 °C, respectively. The wavelength for detection was 475 nm. Calibration curve was generated using the lycopene analytical standard (purity ≥85%, Sigma-Aldrich) in the concentration range of 5 to 50 µmol/kg dichloromethane.

The changes in lycopene concentration in emulsions during storage were regressed with kinetic equation of both zero- and first-order models. Because first-order kinetic model showed better correlation (greater correlation coefficient (*r*^2^)) than zero-order kinetic model, the degradation rate constant (*k*) of lycopene in the emulsions was calculated assuming the following first-order exponential decay model seen in Equation (1):

(1) $$C_{t}=C_{0}\cdot e^{-k\cdot t}$$

where *C*_0_ is the initial lycopene concentration (mmol/kg emulsion) and *C_t_* is the lycopene concentration remaining at time *t* (day). To determine the concentration of lycopene remaining, sampling was carried out at predetermined time intervals: 0, 0.5, 1, 3, 5, 7, and 14 day. The value of *k* was determined from linear regression through the plot of $\ln\left(C_{t}/C_{0}\right)$ versus *t*.

### 2.5. DPPH Radical Scavenging Activity

Radical scavenging activity of TBHQ and lauryl gallate was measured according to the method of Blois [30] with slight modification, using ascorbic acid as the positive control. TBHQ or lauryl gallate were first dissolved in dimethyl sulfoxide (DMSO) to 1 mmol/L and then diluted with distilled/deionized water to 0.1 mmol/L. Sample solution (50 μL) was added 150 μL of 0.1 mM solution of DPPH (2, 2-diphenyl-1-picrylhydrazyl) that was prepared in ethanol. This solution was vigorously vortexed and incubated in dark. After 30 mins, the absorbance was measured at 515 nm against blank sample. The DPPH radical scavenging effect was calculated using the following equation (Equation (2)).

DPPH radical scavenging activity (%) = [(*A*_b_ − *A*_s_)/*A*_b_] × 100 (2)

*A*_b_ is the absorbance of blank sample and *A*_s_ is the absorbance of the sample.

### 2.6. Ferric Reducing Antioxidant Power (FRAP) Assay

The FRAP assay was performed according to a previous method [31]. Stock solutions included 300 mM sodium acetate buffer (pH 3.6), 10 mM 2, 4, 6-tris(2-pyridyl)-1, 3, 5-tri-azine (TPTZ) solution in 40 mM hydrochloric acid, and 20 mM iron (III) chloride solution. FRAP solution was prepared by mixing 10 mL of sodium acetate buffer, 1 mL of TPTZ solution, and 1 mL of iron (III) chloride solution. the mixed solution was incubated at 37 °C in a water bath. The sample solution (20 μL) prepared in DPPH assay was mixed with 150 μL of FRAP solution. The absorbance was measured at 593 nm immediately using the spectrophotometer. the FRAP value was recorded with the following equation (Equation (3))

FRAP value (%) = [(*A*_s_ − *A*_b_)/(*A*_c_ − *A*_b_)] × 2 (3)

*A*_c_ is the absorbance of the positive control, reacted with ascorbic acid (20 μL) and the FRAP solution (150 μL). *A*_s_ is the absorbance of the sample and *A*_b_ is the absorbance of blank sample.

### 2.7. Statistical Analysis

All the experiments were performed in triplicate and the data are expressed as mean ± standard deviation. The Chow test was performed to test the equality of coefficients (degradation rate constant (*k*)) of different linear regressions using SPSS software version 20.0 (IBM Corp., Armonk, NY, USA).

## 3. Results and Discussion

To diminish positive or negative impact of the micelles formed from unabsorbed emulsifiers on the stability of the emulsions and lycopene encapsulated therein [32,33], The MECs of S10, S20, and S100 were 3.17, 2.93, and 1.00 mM, respectively. MECs of S10, S20, and S100 were determined following the procedure described in ‘Materials and Methods’. Since MEC was the minimum emulsifier concentration require to produce the emulsion having the smallest droplet under the homogenization conditions applied in this study, when an emulsion is prepared at the MEC of S10, S20, and S100, all the emulsifier molecules could be found at the interface between oil and water and the amount of unabsorbed emulsifiers might be negligibly small. When emulsions were prepared at the MECs of S10, S20, and S100, the droplet diameters of the freshly prepared emulsions were all similar (*d*_43_ = 0.35, 0.37, and 0.35 μm for S10-, S20-, and S100-stabilized emulsions, respectively), and they changed little during a 14-day storage period. When the mean oil droplet diameter of an emulsion and its oil volume fraction are known, the total number of oil droplets can be roughly calculated by calculating the oil volume of an oil droplet. Also, the total interfacial area of an emulsions can be calculated with the total number of oil droplets and the interfacial area of an oil droplet. Since emulsions prepared in this study had the same oil volume fraction and a similar droplet size, the total interfacial areas of emulsions calculated using their mean oil droplet diameter (0.35, 0.37, and 0.35 μm for S10-, S20-, and S100-stabilized emulsions, respectively) and oil volume fraction (5%, *w*/*w*). Then, the emulsifier concentration per unit of oil droplet surface area could be approximately calculated by dividing the emulsifier concentrations in emulsions by the total interfacial areas of emulsions. The values of the emulsifier concentration per unit of oil droplet surface area for S10-, S20-, and S100-stabilized emulsions were 1.95, 1.92, and 0.63 μmol/m^2^, respectively. Considering these values, emulsions stabilized with S10 and S20 had similar interfacial densities but showed a significantly higher interfacial densities than S100-stabilized emulsions (*p* < 0.05). Emulsions generally have three phases: an oil phase, an aqueous phase, and an interfacial membrane (or region). The interfacial membrane of emulsions consists of an inner layer formed with the hydrophobic tails of emulsifiers and an outer layer formed with their hydrophilic heads and the characteristics of inner and outer layers can determine the interfacial characteristics (such as an interfacial film thickness, and interfacial tension and rheology). The length of the hydrophobic tails of emulsifiers is same and their hydrophilic group length (or size) differs each other, the characteristics of the interfacial membrane may be strongly influenced by the outer layer formed with the hydrophilic heads of emulsifiers. Because S100 has 5- and 10-times greater number of oxyethylene groups than S20 and S10, respectively, although the thickness of outer layer was not exactly proportional to a number of oxyethylene groups, the outer layer of the interfacial film formed with S100 may be thicker interfacial membrane than S20-stabilized emulsions and it could be much thicker interfacial membrane than S10-stabilized one.

### 3.1. Effect of pH on the Stability of Lycopene in Emulsions

In an emulsion system, the interfacial membrane formed with emulsifiers isolates lipids and lipophilic compounds from the aqueous phase that contains compounds that are able to oxidize lipids or to degrade lipophilic compounds. The characteristics of interfacial films could be important in controlling the rate of lipid oxidation and the rate of degradation of functional lipophilic compounds encapsulated in the emulsion droplets because the interactions between lipophilic and hydrophilic compounds occur at the emulsion droplet surfaces. According to previous reports [16,34,35], the influence of interfacial characteristics on the lipid oxidation of emulsified oils and the chemical stability of lipophilic compounds encapsulated therein is in no way negligible.

**Figure 2:**
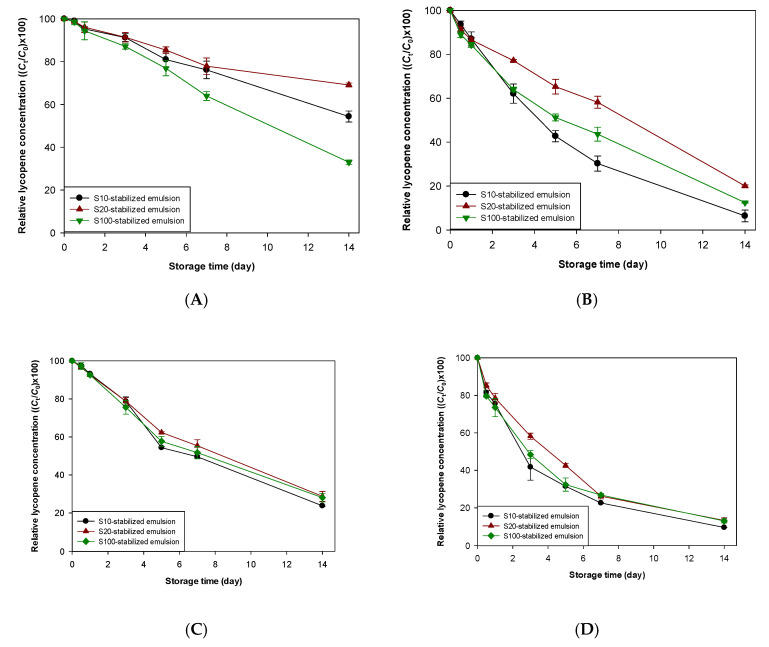
Change in lycopene concentration over storage time in emulsions stored at 25 °C at pH 7 (**A**,**C**) and 3 (**B**,**D**) in the absence (**A**,**B**) and presence (**C**,**D**) of water-soluble radicals. *C*_0_, initial lycopene concentration in emulsion; *C*_t_, lycopene concentration in emulsion at *t* day of storage.

The effect of emulsion pH on the chemical stability of lycopene was evaluated by adjusting the emulsion pH to 3 and 7 (Figure 2A,B). Lycopene in emulsions degraded over time and lycopene degraded much more rapidly at pH 3 than at pH 7 (Figure 2A,B and Figure S1, and Table 1). The present findings agreed well with previous experiments that the pH of the emulsion significantly influenced the stability of lycopene, with the rapid degradation occurring at acidic pH values [36]. Interestingly, unlike our expectations, lycopene in the emulsion at pH 7 steadily degraded during storage (Figure 2A). Although heat and/or light could induce carotenoid degradation, since emulsions were stored in darkness at 25 °C, the lycopene in emulsions stored at pH 7 may degrade through pathways that are not induced by heat or light. The heat generated during homogenization could be a potential reason for the degradation of lycopene in emulsions at pH 7. Although nitrogen purging was carried out during the emulsion preparation to minimize the lycopene degradation induced by oxygen, the oxygen not completely removed from the aqueous phase could explain the lycopene degradation at pH 7. When carotenoids are exposed to an acidic environment, their protonation (carotenoid carbocations) can easily occur [37], and the formed protonated carotenoids undergo a continual degradation process. As shown in Figure 2 and Table 1, our findings agreed with a previous study reporting that the stability of β-carotene in emulsions was inversely related to the size of the hydrophilic groups of emulsifiers to create emulsions [16]. Since the S20-stabilized emulsion has a similar interfacial density to that of the S10-stabilized one, but the number of oxyethylene groups in S20 is a 2-times greater than that of S10, it appeared that the stability of lycopene in an emulsion was proportionally related to the number of oxyethylene groups of hydrophilic groups in emulsifiers used to create emulsions (Table 1). However, this statement could not be directly applied to explain the lycopene degradation pattern in S100-stabilized emulsions. Although the interfacial membrane of the S100-stabilized emulsion was thickest among the emulsions studied in this work, the emulsifier density at the interfacial membrane of S100-stabilized emulsion was lowest between all emulsion samples. The *k* value of lycopene for the S100-stabilized emulsion was highest among emulsions at neutral pH, but was the S100-stabilized emulsion was between S10 and S20-stabilized emulsions. Therefore, considering the interfacial characteristics, such as the number of oxyethylene groups of hydrophilic groups in emulsifiers and the emulsifier concentration (density) at the interfacial films, of all emulsions, it would be better to determine the systematic relationship between the characteristics of the interfacial film and its ability to improve lycopene stability against acid-promoted degradation, which is unclear.

### 3.2. Impact of Free Radicals on Lycopene Stability in Emulsions

As described above, although heat, light, and oxygen are the major factors in carotenoid decomposition, these radicals that originate from lipid molecules may be involved in carotenoid degradation, particularly in emulsions [10]. Several carotenoid degradation mechanisms by radical compounds include electron transfer [38], hydrogen abstraction [39], and carotenoid-radical adduct formation [40]. In emulsion systems, if these radical compounds originating from lipid molecules are still oil-soluble, since radical compounds originating from lipid molecules and oil-soluble functional compounds are located together in emulsion droplets, these radical compounds could accelerate the degradation of oil-soluble bioactives by direct interaction between them. However, if radical compounds are water-soluble, the interfacial membrane may prevent the direct attack of water-soluble radical compounds on lipid molecules and/or lipophilic functional compounds in oil droplets. This suggests that the rate and extent of the degradation of lipophilic functional compounds in emulsions could vary depending on how the interfacial film interrupts the interaction between oil-soluble and water-soluble compounds. Therefore, using AAPH, which decomposes in water and becomes a water-soluble radical compound, the effect of radical compounds on lycopene stability in emulsions with various interfacial properties was evaluated (Figure 2C,D). When emulsions contain water-soluble prooxidants, their lipid oxidation mainly occur at the interfacial region of oil droplets [41,42]. Similar to lipid oxidation of emulsions, the oil-soluble compounds encapsulated in oil droplets can be decomposed at the interfacial region of oil droplets when emulsions contain the compounds capable of promoting the degradation of lipophilic compounds.

At neutral pH, the stability of lycopene encapsulated in the S10-stabilized emulsion against radicals was lower than that in other emulsions. When emulsions were stored at acidic pH, since lycopene in the S10-stabilized emulsion was degraded more rapidly than in the S20- and S100-stabilized emulsions, it appeared that the interfacial membrane formed with emulsifiers having the large (large number of oxyethylene groups in this study) hydrophilic groups prevented the attack of radicals on lycopene more effectively than one formed with emulsifiers having the small (samll number of oxyethylene groups in this study) hydrophilic groups. However, *k* values between emulsions stored at acidic pH was not significantly different (Table 1). Comparing the degree of the *k* value increment in the S10-stabilized emulsion by radical addition with that (approximately 3.3) in the S20-stabilized emulsion at neutral and acidic pH, it appeared the interfacial membrane formed with emulsifiers having the small hydrophilic groups prevented the attack of radicals on lycopene more effectively than one formed with emulsifiers having the large hydrophilic groups. Comparing the *k* values of emulsions containing radicals and considering the degrees of *k* value increment in emulsions after radical addition, it is not easy to interpret the effect of radicals on the stability of lycopene encapsulated in emulsions with various interfacial characteristics. Considering the data obtained here, the number of oxyethylene groups of emulsifiers and the emulsifier concentration (density) at the interfacial films would not be a major determinant controlling the chemical stability of lycopene in emulsions against water-soluble radicals. The possible reason for this statement is that the different abilities of interfacial membranes could be negligible because of the complicated mechanism of radical-induced lycopene degradation.

### 3.3. Impact of Antioxidants on Lycopene Stability in Emulsions

**Figure 3:**
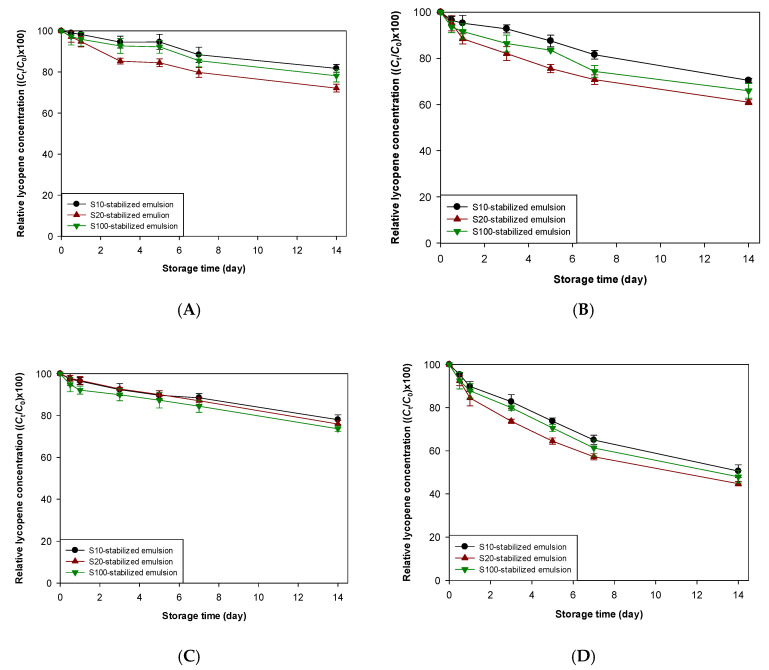
Effect of TBHQ (**A**,**B**) and lauryl gallate (**C**,**D**) on the stability of lycopene concentrations over storage in emulsions stored at 25 °C at pH 7 (**A**,**C**) and 3 (**B**,**D**). *C*_0_, initial lycopene concentration in emulsion; *C*_t_, lycopene concentration in emulsion at *t* day of storage.

**Figure 4:**
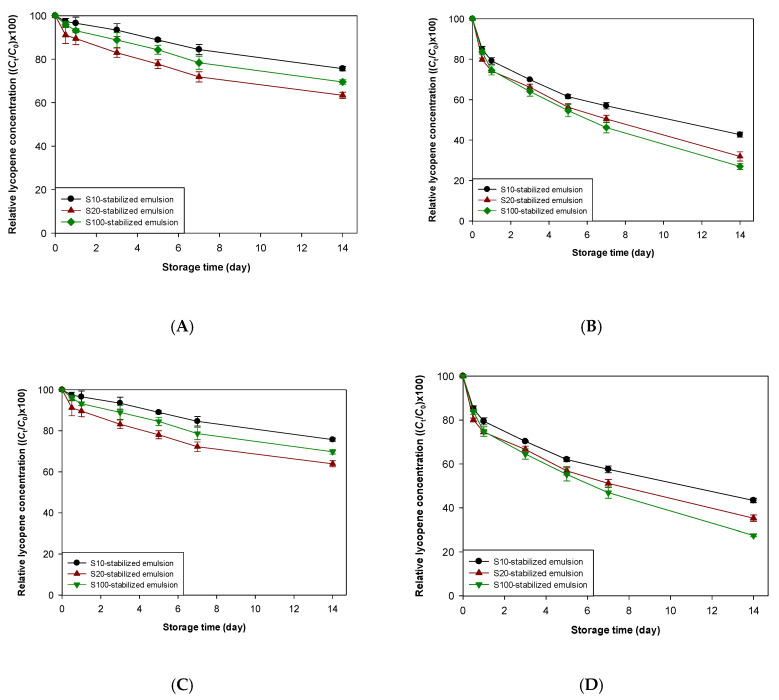
Effect of TBHQ (**A**,**B**) and lauryl gallate (**C**,**D**) on the stability of lycopene concentrations over storage in emulsions stored at 25 °C at pH 7 (**A**,**C**) and 3 (**B**,**D**) in the presence of water-soluble radicals. *C*_0_, initial lycopene concentration in emulsion; *C*_t_, lycopene concentration in emulsion at *t* day of storage.

As shown in Figure 2, the properties of the interfacial membrane of emulsions affected the chemical stability of lycopene encapsulated therein but sometimes did not affect the chemical stability of lycopene under certain environmental stresses. Similar to these observations, the activities of antioxidants incorporated into emulsions studied in this work could be affected by the interfacial membranes of emulsions. To evaluate the effectiveness of antioxidants in emulsions with various interfacial membrane properties, two different types of antioxidants, TBHQ (lipophilic antioxidant) and lauryl gallate (amphiphilic antioxidant), were incorporated into emulsions (Figure 3 and Figure 4). The physical locations of TBHQ and lauryl gallate within emulsions may differ notably because of the difference in polarity between TBHQ and lauryl gallate. Since the polarity of antioxidants having ionizable groups is considerably affected by the pH of aqueous phase of emulsions, partitioning of antioxidants having ionizable groups into different phases (aqueous and oil phases, and interfacial region) of emulsions may be greatly affected upon changing the pH [24,43]. Although phenols on TBHQ and lauryl gallate can form phenoxide ions by losing hydrogen ion (proton), p*K*_a_ values for TBHQ and lauryl gallate are 10.8 and 7.9, respectively [44], they were not ionized under the conditions applied in this study. Therefore, partitioning of TBHQ and lauryl gallate was not altered by changing the pH of emulsions and their partitioning into different phases of emulsions at pH 7 was not significantly different that at pH 3. When TBHQ or lauryl gallate is incorporated into emulsion systems, TBHQ, a lipophilic antioxidant, would be more concentrated in the oily core of oil droplets, and lauryl gallate, an amphiphilic antioxidant, would be more concentrated at the interface between the oil and aqueous phases at a relatively high concentration [23,45,46]. In addition to the physical location of TBHQ and lauryl gallate in emulsions, the antioxidant activities of TBHQ and lauryl gallate were determined using DPPH and FRAP assays since the difference in antioxidant activity between TBHQ and lauryl gallate could make the difference in the ability to inhibit lycopene degradation. Lauryl gallate (85.6% of DPPH inhibition) showed higher DPPH radical scavenging activity than TBHQ (70.1%), but TBHQ (1.9) showed almost 2-times stronger ferric ion reducing activity than lauryl gallate (1.0).

Regardless of pH and the presene of water-soluble radicals, the incorporation of antioxidants effectively increased the chemical stability of lycopene encapsulated into emulsions (Table 2 and Table 3, and Figures S2 and S3). At pH 7, the ability of TBHQ to decrease the oxidative degradation of lycopene seemed to be very similar to that of lauryl gallate. S10-stabilized emulsions showed the smallest *k* value between emulsions, and S20-stabilized emulsions showed the greatest *k* value between emulsions. The *k* values of S10- and S20-stabilized emulsions containing TBHQ were 33 and 83%, respectively, of those of respective antioxidant-free emulsions, and the values of S10- and S20-stabilized emulsions containing lauryl gallate were 38 and 68%, respectively, of those of respective antioxidant-free emulsions. When water-soluble radicals were a major cause of the degradation of lycopene encapsulated into emulsions at pH 7, very similar results were also observed. The *k* values of S10- and S20-stabilized emulsions containing TBHQ were 19 and 34% of those of respective antioxidant-free emulsions, and the values of S10- and S20-stabilized emulsions containing lauryl gallate were 19 and 33% of those of respective antioxidant-free emulsions. Comparing S10- and S20-stabilized emulsions (they had a very similar interfacial density), antioxidants, independent of their polarities and DPPH radical scavenging activities, increased the chemical stability of lycopene in emulsions stabilized with emulsifier having a small number of oxyethylene groups in its hydrophilic group more effectively. However, considering all of the *k* values for emulsions stored at pH 7, the relationship between antioxidant effectiveness and interfacial membrane properties was unclear.

The results obtained from emulsions stored at pH 3 were somewhat different from those obtained from emulsions stored at pH 7. When the acid-mediated mechanism was the only lycopene degradation pathway, TBHQ was more effective than lauryl gallate (Table 2 and Table 3, and Figures S2 and S3) and there was no significant difference in *k* value between emulsions containing lauryl gallate (Table 3). Comparing the *k* values of emulsions containing antioxidants with those of antioxidant-free emulsions, since the degree of the decrease in *k* value of emulsions stabilized with emulsifier having a small number of oxyethylene groups in its hydrophilic group was greater than in emulsions stabilized with emulsifier having a large number of oxyethylene groups, antioxidants, regardless of their polarity, worked better in emulsions stabilized with emulsifiers having a small number of oxyethylene groups than in those stabilized with emulsifiers having a large number of oxyethylene groups. TBHQ offered similar antioxidant activity as lauryl gallate when water-soluble radicals were present in emulsions at pH 3. Interestingly, the smallest *k* value was found in the S10-stabilized emulsion, and the largest value was found in the S100-stabilized emulsion, independent of the type of antioxidants present in emulsions. As in the previous study regarding the determination of the effect of the lipophilic antioxidant (TBHQ) on the stabilization of lycopene in emulsions, TBHQ significantly increased lycopene stability at pH 7 but it did not at pH 3 [36]. This could be due to the difference between this and previous studies in the emulsifier characteristics used to create emulsions. The emulsifiers used in the previous study [36] were anionic surfactant, sodium dodecyl sulfate (SDS), and anionic SDS at droplet interfaces which promote the lycopene degradation by attracting cationic transition metals capable of promoting the lycopene degradation at the interfaces.

Since nonpolar free radical scavenging antioxidants partition into emulsion droplets more than polar ones, nonpolar antioxidants are more effective in the prevention and/or inhibition of lipid oxidation and the degradation of lipophilic functional compounds in oil-in-water emulsions [23,45]. According to the previous study [21], among the free radical scavengers, oil soluble antioxidants (α-tocopherol) were more effective in increasing the lycopene stability in emulsions than were surface-active antioxidants (propyl gallate). However, as described above, since antioxidants having suitable hydrophilic-lipophilic balance values can be more concentrated at the interfacial region of emulsions as the main site of lipid oxidation and the degradation of lipophilic functional compounds, amphiphilic antioxidants are more effective than strongly hydrophobic and hydrophilic antioxidants in the prevention and/or inhibition of lipid oxidation and the degradation of lipophilic functional compounds [47,48]. Also, since the radical scavenging activity of TBHQ was lower than that of lauryl gallate, it was expected that lauryl gallate showed a better ability to inhibiting lycopene degradation than TBHQ. However, these data in this study showed that there was no significant difference in the ability of nonpolar and polar antioxidants to protect lycopene from radical-mediated degradation. The most difficult observation to understand was how TBHQ was more effective in stabilizing lycopene from acid-mediated degradation than lauryl gallate. Considering the molecular structures of antioxidants used in this study and their antioxidant mechanism, the inhibition of protonated lycopene could not be a main mechanism for their improvement of the chemical stability of lycopene. As described above, protonated lycopene could degrade throughout the following steps. It is believed that TBHQ and lauryl gallate can break a certain step (or certain steps) of the lycopene degradation chain reaction that starts from protonated lycopene. Considering the molecular structure of lycopene, since intermediates of the lycopene degradation process starting from protonated lycopene must be nonpolar, TBHQ could directly prevent intermediates from going to the next step. However, lauryl gallate could be less effective at preventing these intermediates from going to the next step because the moiety of lauryl gallate has polar antioxidant activity (hydrophilic) and is located at the aqueous phase, not the oil phase. It is not clear how TBHQ and lauryl gallate improved the chemical stability of lycopene against acid-mediated degradation and why TBHQ was more effective than lauryl gallate under this condition.

## 4. Conclusions

The results show that the interfacial characteristics, such as the outer layer formed the hydrophilic group size of emulsifiers and the emulsifier concentration (density) at the interfacial films, seemed to have some effect on the storage stability of lycopene encapsulated into emulsions and that antioxidants played a role in improving the lycopene encapsulated into emulsions. The findings of this work are different from a previous report showing that the degradation rate of β-carotene encapsulated into emulsions was highly affected by the size of hydrophilic groups of emulsifiers used to emulsions [16]. Although it is difficult to generalize the findings of this work to all carotenoids, the properties of the interfacial membranes of emulsions may or may not affect the chemical stability of carotenoids, and the ability of the interfacial film to retard/inhibit carotenoid degradation in emulsions may highly depend on the type of carotenoid. Lipophilic (TBHQ) and amphiphilic (lauryl gallate) antioxidants were found to inhibit the lycopene degradation occurring due to oxidative stress of the aqueous phase. These antioxidants had a similar effectiveness in protecting lycopene in emulsions from radical-mediated degradation, but TBHQ was more effective in inhibiting the degradation of lycopene encapsulated into emulsions at acidic pH than lauryl gallate.

## Figures and Tables

**Table 1:** Effect of pH and water-soluble free radicals on the degradation rate constant (*k*) of lycopene in emulsions.

	Free Radicals	S10			S20			S100		
		*k* (day^−1^)	S.E.	*r* ^2^	*k* (day^−1^)	S.E.	*r* ^2^	*k* (day^−1^)	S.E.	*r* ^2^
pH 7	Absent	^C^ 0.0435 ^b^	0.0015	0.994	^D^ 0.0273 ^c^	0.0021	0.972	^C^ 0.0788 ^a^	0.0051	0.980
	Present	^B^ 0.1048 ^a^	0.0045	0.991	^C^ 0.0894 ^b^	0.0018	0.998	^B^ 0.0923 ^b^	0.0034	0.993
pH 3	Absent	^A^ 0.1956 ^a^	0.0062	0.995	^B^ 0.1086 ^c^	0.0096	0.963	^A^ 0.1431 ^b^	0.0075	0.987
	Present	^A^ 0.1657 ^a^	0.0140	0.966	^A^ 0.1452 ^a^	0.0100	0.977	^A^ 0.1424 ^a^	0.0136	0.956

The values with different capital-letter superscripts in a same column are significantly different by Chow test (*p* < 0.05). The values with different small-letter superscripts in a same row are significantly different by Chow test (*p* < 0.05). S.E. indicates the standard error.

**Table 2:** Effect of pH and water-soluble free radicals on the degradation rate constant (*k*) of lycopene in emulsions containing TBHQ.

	Free Radicals	S10			S20			S100		
		*k* (day^−1^)	S.E.	*r* ^2^	*k* (day^−1^)	S.E.	*r* ^2^	*k* (day^−1^)	S.E.	*r* ^2^
pH 7	Absent	^C^ 0.0145 ^b^	0.0011	0.972	^C^ 0.0227 ^a^	0.0030	0.919	^C^ 0.0169 ^ab^	0.0014	0.969
	Present	^C^ 0.0197 ^b^	0.0010	0.988	^BC^ 0.0301 ^a^	0.0038	0.928	^B^ 0.0251 ^a^	0.0021	0.967
pH 3	Absent	^B^ 0.0244 ^b^	0.0011	0.989	^B^ 0.0339 ^a^	0.0042	0.930	^B^ 0.0282 ^ab^	0.0029	0.951
	Present	^A^ 0.0550 ^b^	0.0068	0.929	^A^ 0.0730 ^ab^	0.0070	0.956	^A^ 0.0866 ^a^	0.0064	0.974

The values with different capital-letter superscripts in a same column are significantly different by Chow test (*p* < 0.05).The values with different small-letter superscripts in a same row are significantly different by Chow test (*p* < 0.05).The bold values indicate the significant difference in *k* values between emulsions containing TBHQ (Table 2) and lauryl gallate (Table 3) under a same environmental stress by Chow test (*p* < 0.05).S.E. indicates the standard error.

**Table 3:** Effect of pH and water-soluble free radicals on the degradation rate constant (*k*) of lycopene in emulsions containing lauryl gallate.

	Free Radicals	S10			S20			S100		
		*k* (day^−1^)	S.E.	*r* ^2^	*k* (day^−1^)	S.E.	*r* ^2^	*k* (day^−1^)	S.E.	*r* ^2^
pH 7	Absent	^C^ 0.0167 ^b^	0.0009	0.985	^C^ 0.0189 ^a^	0.0005	0.996	^D^ 0.0194 ^ab^	0.0017	0.962
	Present	^B^ 0.0196 ^b^	0.0009	0.990	^B^ 0.0296 ^a^	0.0037	0.927	^C^ 0.0248 ^a^	0.0020	0.968
pH 3	Absent	^A^ 0.0482 ^a^	0.0030	0.981	^A^ 0.0558 ^a^	0.0062	0.943	^B^ 0.0513 ^a^	0.0040	0.970
	Present	^A^ 0.0540 ^b^	0.0067	0.929	^A^ 0.0664 ^ab^	0.0076	0.939	^A^ 0.0855 ^a^	0.0062	0.974

The values with different capital-letter superscripts in a same column are significantly different by Chow test (*p* < 0.05).The values with different small-letter superscripts in a same row are significantly different by Chow test (*p* < 0.05). The bold values indicate the significant difference in *k* values between emulsions containing TBHQ (Table 2) and lauryl gallate (Table 3) under a same environmental stress by Chow test (*p* < 0.05). S.E. indicates the standard error.

## Supplementary Materials

The following are available online at https://www.mdpi.com/2304-8158/9/8/971/s1, Figure S1. First-order kinetic decay plots for lycopene in emulsions at pH 7 (A and C) and 3 (B and D) in the absence (A and B) and presence (C and D) of water-soluble free radicals. *C*_0_, initial lycopene concentration in emulsion; *C*_t_, lycopene concentration in emulsion at *t* day of storage. Figure S2. First-order kinetic decay plots for lycopene in emulsions containing TBHQ (A and B) and lauryl gallate (C and D) at pH 7 (A and C) and 3 (B and D). *C*_0_, initial lycopene concentration in emulsion; *C*_t_, lycopene concentration in emulsion at *t* day of storage. Figure S3. First-order kinetic decay plots for lycopene in emulsions containing TBHQ (A and B) and lauryl gallate (C and D) at pH 7 (A and C) and 3 (B and D) in the presence of water-soluble radicals. *C*_0_, initial lycopene concentration in emulsion; *C*_t_, lycopene concentration in emulsion at *t* day of storage.

## References

[1] Olson J.A., Krinsky N.I. (1995). Introduction: The colorful fascinating world of the carotenoids: Important physiologic modulators. FASEB J..

[2] Maiani G., Periago Castón M.J., Catasta G., Toti E., Cambrodón I.G., Bysted A., Granado-Lorencio F., Olmedilla-Alonso B., Knuthsen P., Valoti M. (2009). Carotenoids: Actual knowledge on food sources, intakes, stability and bioavailability and their protective role in humans. Mol. Nutr. Food Res..

[3] Britton G. (1995). Structure and properties of carotenoids in relation to function. FASEB J..

[4] Fiedor J., Burda K. (2014). Potential role of carotenoids as antioxidants in human health and disease. Nutrition.

[5] Di M.P., Kaiser S., Sies H. (1989). Lycopene as the most efficient biological carotenoid singlet oxygen quencher. Arch. Biochem. Biophys..

[6] Miller N.J., Sampson J., Candeias L.P., Bramley P.M., Rice-Evans C.A. (1996). Antioxidants activities of carotenes and xanthophylls. FEBS Lett..

[7] Giovannucci E. (2005). Tomato products, lycopene, and prostate cancer: A review of the epidemiological literature. J. Nutr..

[8] Wilcox J., Catignani G., Lazarus S. (2003). Tomatoes and cardiovascular health. Crit. Rev. Food Sci. Nutr..

[9] Kun Y., Lule U.S., Xiao-Lin A.D. (2006). Lycopene: Its properties and relationship to human health. Food Rev. Int..

[10] Boon C.S., McClements D.J., Weiss J., Decker E.A. (2010). Factors influencing the chemical stability of carotenoids in foods. Crit. Rev. Food Sci. Nutr..

[11] Mao L., Miao S. (2015). Structuring food emulsions to improve nutrient delivery during digestion. Food Eng. Rev..

[12] Neves M.A., Hashemi J., Prentice C. (2015). Development of novel bioactives delivery systems by micro/nanotechnology. Curr. Opin. Food Sci..

[13] Song H.Y., Moon T.W., Choi S.J. (2019). Impact of antioxidant on the stability of β-carotene in model beverage emulsions: Role of emulsion interfacial membrane. Food Chem..

[14] Salvia-Trujillo L., Qian C., Martín-Belloso O., McClements D.J. (2013). Modulating β-carotene bioaccessibility by controlling oil composition and concentration in edible nanoemulsions. Food Chem..

[15] Liu X., Zhang R., McClements D.J., Li F., Liu H., Cao Y., Xiao H. (2018). Nanoemulsion-based delivery systems for nutraceuticals: Influence of long-chain triglyceride (LCT) type on in vitro digestion and astaxanthin bioaccessibility. Food Biophys..

[16] Song H.Y., Moon T.W., Choi S.J. (2018). Storage stability of β-carotene in model beverage emulsions: Implication of interfacial thickness. Eur. J. Lipid Sci. Technol..

[17] McClements D.J. (2013). Nanoemulsion-based oral delivery systems for lipophilic bioactive components: Nutraceuticals and pharmaceuticals. Ther. Deliv..

[18] Qian C., Decker E.A., Xiao H., McClements D.J. (2012). Physical and chemical stability of β-carotene-enriched nanoemulsions: Influence of pH, ionic sterngth, temperature, and emulsifier type. Food Chem..

[19] Qian C., Decker E.A., Xiao H., McClements D.J. (2012). Inhibition of β-carotene degradation in oil-in-water nanoemulsions: Influence of oil-soluble and water-soluble antioxidants. Food Chem..

[20] Guan Y., Wu J., Zhong Q. (2016). Eugenol improves physical and chemical stabilities of nanoemulsions loaded with β-carotene. Food Chem..

[21] Bou R., Boon C., Kweku A., Hidalgo D., Decker E.A. (2011). Effect of different antioxidants on lycopene degradation in oil-in-water emulsions. Eur. J. Lipid Sci. Technol..

[22] Frankel E.N., Huang S.-W., Kanner J., German J.B. (1994). Interfacial phenomena in the evaluation of antioxidants: Bulk oils vs. emulsions. J. Agric. Food Chem..

[23] Huang S.-W., Frankel E.N., Aeschbach R., German J.B. (1997). Partition of selected antioxidants in corn oil-water model systems. J. Agric. Food Chem..

[24] Jacobsen C., Schwarz K., Stöckmann H., Meyer A.S., Alder-Nissen J. (1999). Partioning of selected antioxidants in mayonnaise. J. Agric. Food Chem..

[25] Losada-Barreiro S., Bravo-Díaz C., Paiva-Martins F., Romsted L.S. (2013). Maxima in antioxidant distributions and efficiencies with increasing hydrophobicity of gallic acid and its alkyl esters. The pseudophase model interpretation of the “cutoff effect”. J. Agric. Food Chem..

[26] Ferreira I., Costa M., Losada-Barreiro S., Paiva-Martins F., Bravo-Díaz C. (2018). Modulating the interfacial concentration of gallates to improve the oxidative stability of fish oil-in-water emulsions. Food Res. Int..

[27] Han S.W., Song H.Y., Moon T.W., Choi S.J. (2018). Influence of emulsion interfacial membrane characteristics on Ostwald ripening in a model emulsion. Food Chem..

[28] Niki E. (1990). Free radical initiators as source of water- or lipid-soluble peroxyl radicals. Methods Enzymol..

[29] Barba A.O., Hurtado M.C., Mata M.S., Ruiz V.F., de Tejada M.L.S. (2006). Application of a UV–vis detection-HPLC method for a rapid determination of lycopene and β-carotene in vegetables. Food Chem..

[30] Blois M.S. (1958). Antioxidant determination by the use of a stable free radical. Nature.

[31] Benzie I.F.F., Strain J.J. (1996). The ferric reducing ability of plasma (FRAP) as a measure of “antioxidant power”: The FRAP assay. Anal. Biochem..

[32] McClements D.J. (1994). Ultrasonic determination of depletion flocculation in oil-in-water emulsions containing a non-ionic surfactant. Colloid Surf. A Physicochem. Eng. Asp..

[33] Cho Y.-J., McClements D.J., Decker E.A. (2002). Ability of surfactant micelles to alter the physical location and reactivity of iron in oil-in-water emulsion. J. Agric. Food Chem..

[34] Berton-Carabin C.C., Ropers M.-H., Genot C. (2014). Lipid oxidation in oil-in-water emulsions: Involvement of the interfacial layer. Compr. Rev. Food. Sci. Food Saf..

[35] Lee H.Y., Song H.Y., Choi S.J. (2019). Lipid hydroperoxide decomposition in model emulsions stabilized with emulsifiers having various sizes of hydrophilic heads. Food Sci. Biotechnol..

[36] Boon C.S., McClements D.J., Weiss J., Decker E.A. (2009). Role of iron and hydroperoxides in the degradation of lycopene in oil-in-water emulsions. J. Agric. Food Chem..

[37] Jeevarajan A.S., Wei C.-C., Kispert L.D. (1994). Geometrical isomerization of carotenoids in dichloromethan. J. Chem. Soc. Perkin Trans. 2.

[38] Young A.J., Lowe G.M. (2001). Antioxidant and prooxidant properties of carotenoids. Arch. Biochem. Biophys..

[39] Woodall A.A., Lee S.W.-M., Weesie R.J., Jackson M.J., Britton G. (1997). Oxidation of carotenoids by free radicals: Relationship between structure and reactivity. Biochim. Biophys. Acta.

[40] Liebler D.C., McClure T.D. (1996). Antioxidant reactions of β-carotene: Identification of carotenoid-radical adducts. Chem. Res. Toxicol..

[41] Silvestre M.P.C., Chaiyasit W., Brannan R.G., McClements D.J., Decker E.A. (2000). Ability of surfactant headgroup size to alter lipid and antioxidant oxidation in oil-in-water emulsions. J. Agric. Food Chem..

[42] Waraho T., McClements D.J., Decker E.A. (2011). Mechanisms of lipid oxidation in food dispersions. Trends Food Sci. Technol..

[43] McClements D.J., Decker E.A. (2000). Lipid oxidation in oil-in-water emulsions: Impact of molecular environment on chemical reactions in heterogeneous food systems. J. Food Sci..

[44] Makahleh A., Saad B., Bari M.F., Shahidi F. (2015). Synthetic phenolics as antioxidants for food preservation. Handbook of Antioxidants for Food Preservation.

[45] Huang S.-W., Hopia A., Schwarz K., Frankel E.N., German J.B. (1996). Antioxidant activity of α-tocopherol and trolox in different lipid substrates: Bulk oils vs. oil-in-water emulsions. J. Agric. Food Chem..

[46] Huang S.-W., Frankel E.N., Schwarz K., Aeschbach R., German J.B. (1996). Antioxidant activity of carnosic acid and methyl carnosate in bulk oils and oil-in-water emulsions. J. Agric. Food Chem..

[47] Freiría-Gándara J., Losada-Barreiro S., Paiva-Martins F., Bravo-Díaz C. (2018). Enhancement of the antioxidant efficiency of gallic acid derivatives in intact fish oil-in-water emulsions through optimization of their interfacial concentrations. Food Funct..

[48] Costa M., Losada-Barreiro S., Bravo-Díaz C., Vicente A.A., Monteiro L.S., Paiva-Martins F. (2020). Influence of AO chain length, droplet size and oil to water ratio on the distribution and on the activity of gallates infish oil-in-water emulsified systems: Emulsion and nanoemulsion comparison. Food Chem..

