# Supplementary material for: Improving the Stability of Lycopene from Chemical Degradation in Model Beverage Emulsions: Impact of Hydrophilic Group Size of Emulsifier and Antioxidant Polarity
Source: Foods. 2020 Jul 22;9(8):971. doi: 10.3390/foods9080971 (PMC7466299; doi:10.3390/foods9080971)
Supplement: Supplementary file 1 [file foods-09-00971-s001.pdf]

Supplementary figures

Figure S1. First-order kinetic decay plots for lycopene in emulsions at pH 7 (A and C) and 3 (B and D) in the absence (A and B) and presence (C and D) of water-soluble free radicals. C_0, initial lycopene concentration in emulsion; C_t, lycopene concentration in emulsion at t day of storage.

Figure S2. First-order kinetic decay plots for lycopene in emulsions containing TBHQ (A and B) and lauryl gallate (C and D) at pH 7 (A and C) and 3 (B and D). C_0, initial lycopene concentration in emulsion; C_t, lycopene concentration in emulsion at t day of storage.

Figure S3. First-order kinetic decay plots for lycopene in emulsions containing TBHQ (A and B) and lauryl gallate (C and D) at pH 7 (A and C) and 3 (B and D) in the presence of water-soluble radicals. C_0, initial lycopene concentration in emulsion; C_t, lycopene concentration in emulsion at t day of storage.

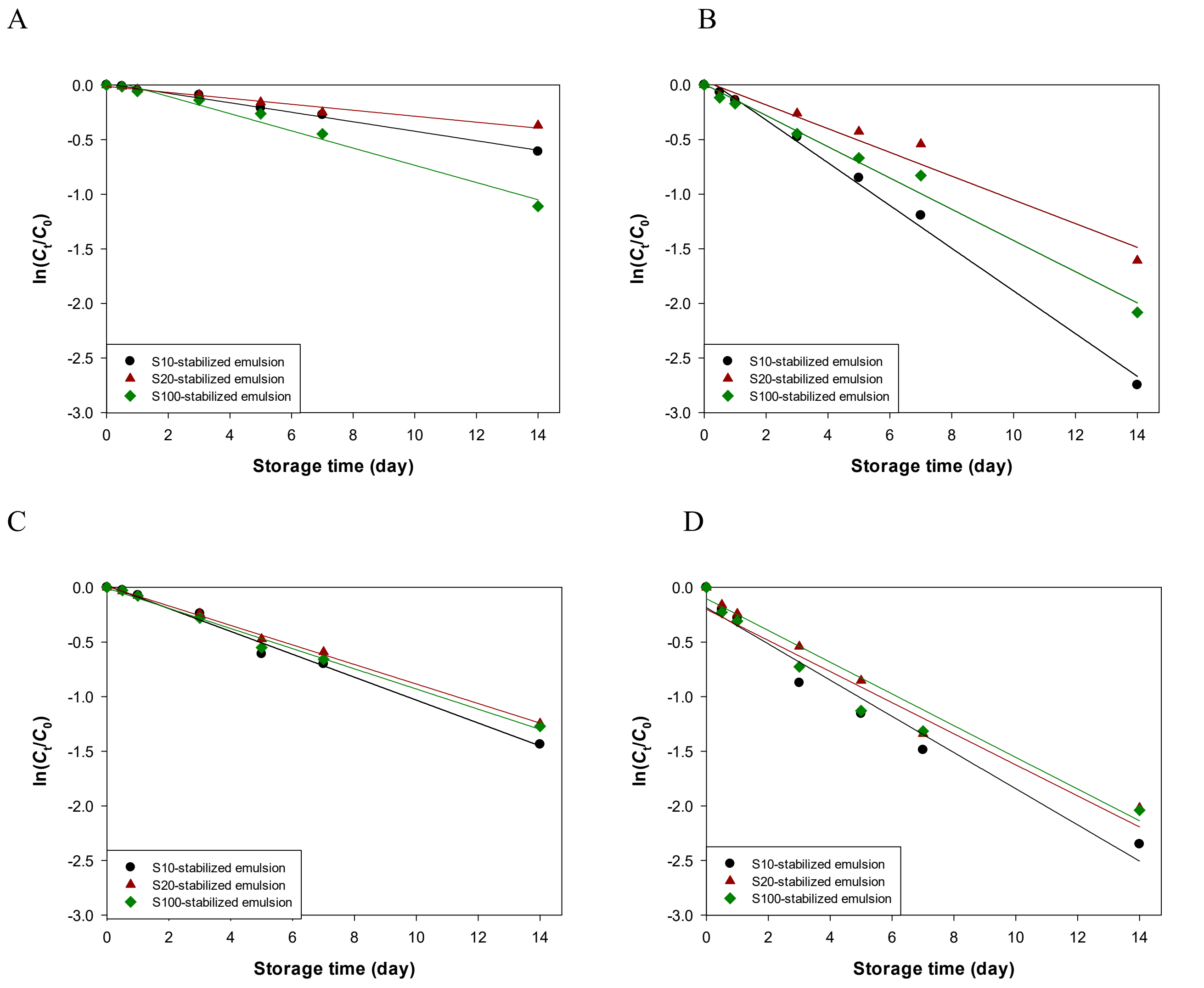

Figure S1. First-order kinetic decay plots for lycopene in emulsions at pH 7 (A and C) and 3 (B and D) in the absence (A and B) and presence (C and D) of water-soluble free radicals. C_0, initial lycopene concentration in emulsion; C_t, lycopene concentration in emulsion at *t* day of storage.

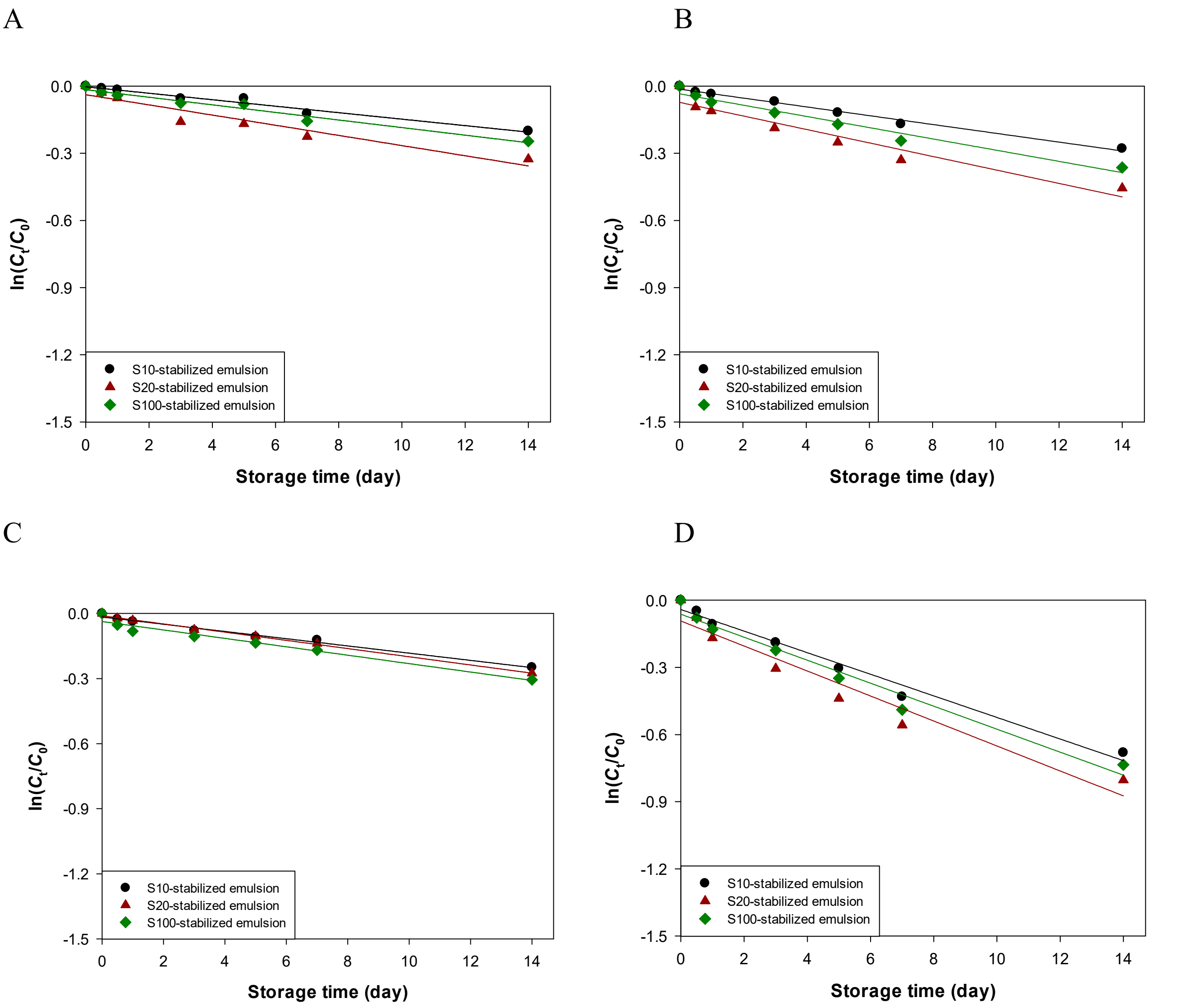

Figure S2. First-order kinetic decay plots for lycopene in emulsions containing TBHQ (A and B) and lauryl gallate (C and D) at pH 7 (A and C) and 3 (B and D). C_0, initial lycopene concentration in emulsion; C_t, lycopene concentration in emulsion at t day of storage.

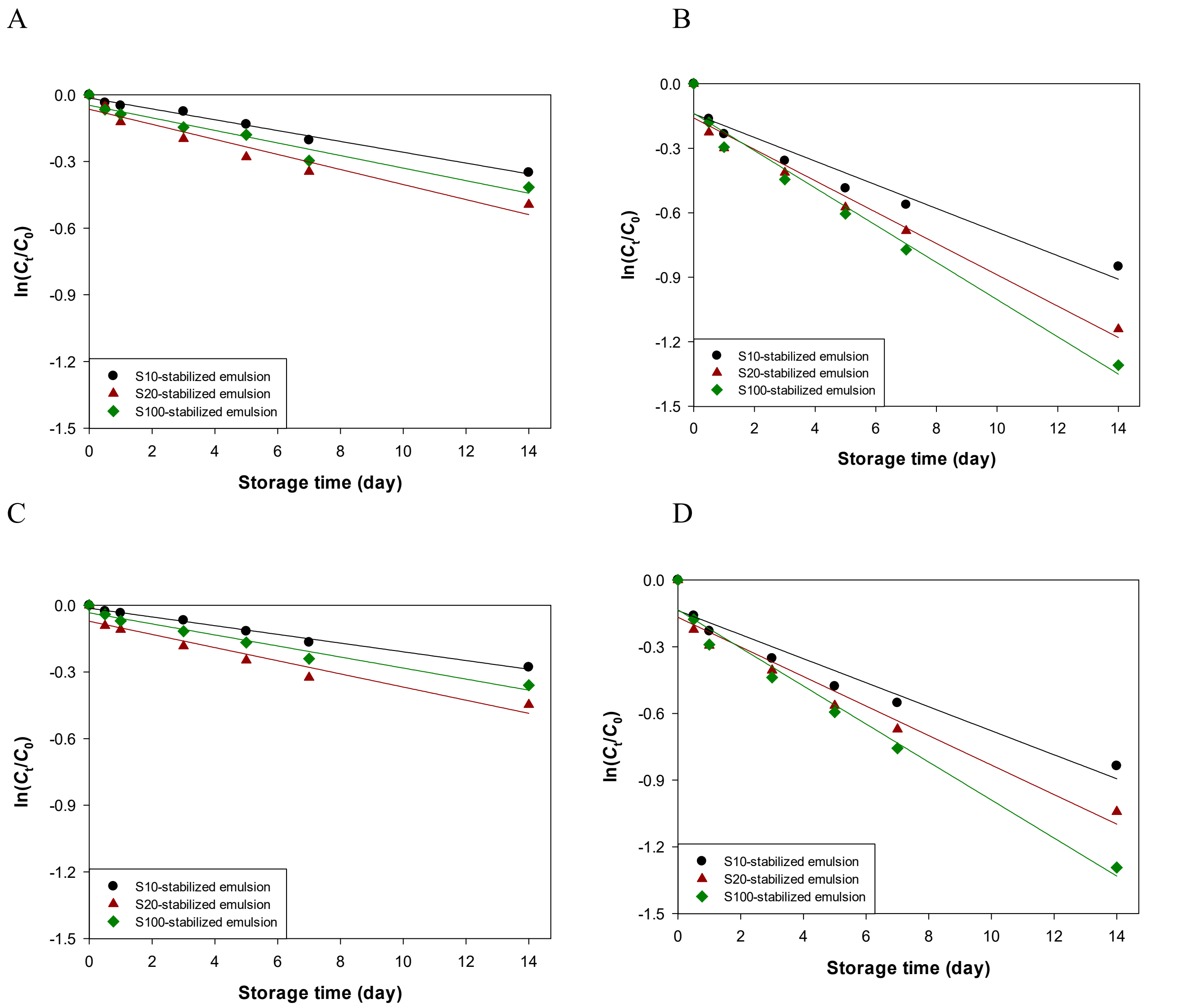

Figure S3. First-order kinetic decay plots for lycopene in emulsions containing TBHQ (A and B) and lauryl gallate (C and D) at pH 7 (A and C) and 3 (B and D) in the presence of water-soluble radicals. C_0, initial lycopene concentration in emulsion; C_t, lycopene concentration in emulsion at *t* day of storage.
